# Non-Destructive Integrity Assessment of Austenitic Stainless-Steel Membranes via Magnetic Property Measurements

**DOI:** 10.3390/ma18122898

**Published:** 2025-06-19

**Authors:** Haeng Sung Heo, Jinheung Park, Jehyun You, Shin Hyung Rhee, Myoung-Gyu Lee

**Affiliations:** 1 Decarbonization Ship R&D Team, Future Energy R&D Center, Hanwha Ocean, Seoul 04527, Republic of Korea; hsheo@hanwha.com; 2 Department of Naval Architecture and Ocean Engineering, Seoul National University, Seoul 08826, Republic of Korea; 3 Department of Materials Science and Engineering & RIAM, Seoul National University, Seoul 08826, Republic of Korea; 4 Department of Integrated Systems Engineering, The Ohio State University, Columbus, OH 43210, USA; 5 Research Institute of Marine Systems Engineering, Seoul National University, Seoul 08826, Republic of Korea

**Keywords:** membrane-type tank, inspection, strain-induced martensitic transformation (SIMT), magnetic non-destructive testing (M-NDT)

## Abstract

This study proposes a novel non-destructive methodology for assessing structural integrity in liquefied natural gas (LNG) carrier cargo containment systems (CCSs), addressing limitations of conventional inspection techniques like visual inspection and vacuum box testing. The method leverages strain-induced martensitic transformation (SIMT) in austenitic stainless steel (SUS304L), widely used in CCS membranes, quantifying magnetic permeability increase via a Feritscope to evaluate deformation history and damage. To analyze SUS304L SIMT behavior, uniaxial tensile (UT) and equi-biaxial tensile (EBT) tests were conducted, as these stress states predominate in CCS membranes. Microstructural evolution was examined using X-ray diffraction (XRD) and electron backscatter diffraction (EBSD), allowing a quantitative assessment of the transformed martensite volume fraction versus plastic strain. Subsequently, Feritscope measurements under the same conditions were calibrated against the XRD-measured martensite volume fraction for accuracy. Based on testing, this study introduces three complementary Feritscope approaches for evaluating CCS health: outlier detection, quantitative damaged area analysis, and time-series analysis. The methodology integrates data-driven quantitative assessment with conventional qualitative inspection, enhancing safety and maintenance efficiency.

## 1. Introduction

The cargo containment system (CCS) of liquefied natural gas (LNG) carriers operates under extreme service conditions, most notably cryogenic temperatures reaching −163 °C [1]. Austenitic stainless steel, particularly SUS304L, is widely employed in critical CCS components such as corrugated membranes and pump towers due to its outstanding mechanical properties at low temperatures and exceptional formability [2]. This material retains high ductility without embrittlement even under cryogenic conditions, allowing it to withstand sloshing impact loads while maintaining structural integrity [3]. Furthermore, its excellent formability facilitates the fabrication of corrugated structures, which are essential for accommodating thermal contraction. Despite being designed with sufficient safety margins and considering structural loads, damage to SUS304L components, including membranes and pump towers, has frequently been reported. Such damage is presumed to stem from uncertainties in sloshing loads, structural and fluid analyses, and experimental validation, potentially leading to unexpected failures [3,4].

To ensure the safety of LNG carriers and mitigate the risks of maritime accidents and environmental pollution, the International Maritime Organization (IMO) and the International Association of Classification Societies (IACS) mandate periodic inspections every five years in accordance with the Safety of Life at Sea (SOLAS) convention, particularly its regulations concerning survey and certification, and in line with the technical standards set forth in the International Code for the Construction and Equipment of Ships Carrying Liquefied Gases in Bulk (IGC Code). These inspections serve as a critical measure for verifying structural integrity and ensuring the continued safe operation of LNG carriers [5]. Ships and offshore structures, particularly liquefied natural gas carriers (LNGCs), operate in a unique and challenging maritime environment, making repair and maintenance significantly more difficult compared to land-based structures. Therefore, precise detection and assessment of structural damage during periodic inspections are directly linked to operational safety.

**Figure 1:**
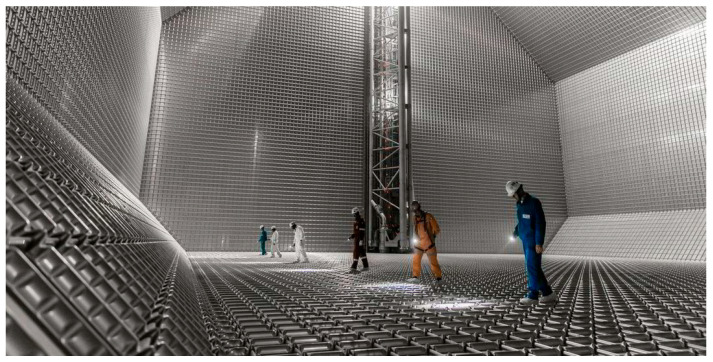
Visual inspection of the primary barrier in a membrane-type LNGC cargo tank.

To enhance the accuracy and efficiency of these inspections, research utilizing various advanced technologies is actively underway. Lin et al. [6] provided a comprehensive review of hull inspection technologies, presenting the development trends of various inspection techniques. Tu et al. [7] proposed a non-destructive testing method using terahertz waves to inspect defects in protective coatings. Veruz et al. [8] and Andersen et al. [9] conducted research on automating underwater hull and offshore structure inspections using computer vision and artificial intelligence (AI). Vázquez et al. [10] emphasized the need for a periodic inspection program for preventive maintenance of offshore monopiles. Alizadeh et al. [11] proposed a risk-based underwater inspection framework for structural integrity management of aging fixed offshore platforms. The current inspection regime mandated by the IMO primarily comprises visual inspections and vacuum box testing. As shown in Figure 1, visual inspections rely heavily on the inspector’s experience and eyesight to identify deformations, cracks, and other forms of damage on the membrane surface.

**Figure 2:**
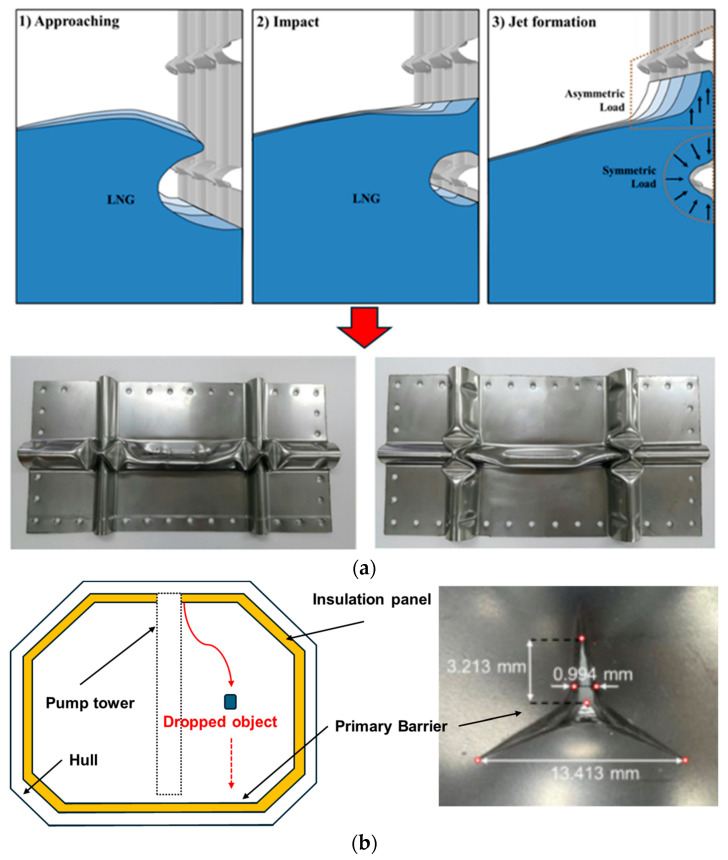
Illustrative case studies simulating common damage mechanisms on the primary membrane of LNG CCS: (**a**) an example of collapse-type failure in corrugations, which can occur due to sloshing loads [19]; (**b**) penetration damage to the primary barrier resulting from the impact of a dropped object, such as a tool or bolt falling from a pump tower due to operational or installation error [20].

However, this method is inherently subjective, as the results may vary depending on the inspector’s judgment. Furthermore, visual inspections pose challenges in quantitatively assessing damage, making it difficult to detect minor or early-stage defects. Following the visual inspection, a vacuum box test is conducted to assess the membrane’s leak-tightness. This method quantitatively detects leaks by identifying pressure variations caused by compromised membrane integrity due to cracks. However, a fundamental limitation of this approach is that it can only detect defects after cracks have already formed, making it ineffective for identifying the onset of crack initiation. Consequently, the existing inspection methods are insufficient for the precise, quantitative assessment of minor or incipient damage, potentially leading to the overlooking of risk factors that could develop into catastrophic failures. Furthermore, as observed in major membrane failure case studies (Figure 2), large-scale damage can be relatively easily identified through visual inspections. However, accurately quantifying its structural impact on adjacent areas remains a significant challenge, complicating the determination of the optimal repair scope. Therefore, to ensure the structural integrity and operational safety of LNG carriers, it is crucial to develop advanced inspection techniques capable of accurately detecting minor damage at an early stage and quantitatively assessing the extent of the affected areas. To achieve this objective, non-destructive testing (NDT) techniques, which can detect and evaluate defects without causing further damage to the structure, are essential. While various non-destructive testing methods exist, including liquid penetrant testing, magnetic particle testing, ultrasonic testing, radiographic testing, and eddy current testing, this study focuses on magnetic non-destructive testing (M-NDT) by leveraging the strain-induced martensitic transformation (SIMT) phenomenon in SUS304L, a commonly used material for LNG carrier cargo containment membranes. As a precursor, Jurkovič et al. [12] evaluated the damage of ship propeller blades after long-term operation using M-NDT. This is noteworthy as it utilizes M-NDT techniques, similar to the approach proposed in this study. Furthermore, the application of M-NDT highlights the growing interest in leveraging magnetic properties for structural health monitoring in the maritime industry. SUS304L exhibits a unique characteristic where it undergoes a phase transformation from austenite to martensite, a ferromagnetic phase, under plastic deformation. M-NDT methods can thus be used to indirectly assess the plastic deformation history and potential damage by measuring the changes in magnetic properties.

SUS304L is known to exist as a metastable face-centered cubic (fcc) austenitic phase at both room and cryogenic temperatures, undergoing a phase transformation into body-centered tetragonal (bct) martensite under plastic deformation, a phenomenon referred to as SIMT. Since martensite exhibits greater mechanical strength than austenite, SIMT contributes to superior hardening behavior, enhancing the mechanical performance. To better understand and utilize this phenomenon, numerous researchers have conducted studies on SIMT behavior. Ryoo et al. [13] identified an inverse correlation between nickel content and SIMT kinetics in austenitic stainless steels, indicating that higher nickel concentrations suppress martensitic transformation. Sunil et al. [14] experimentally demonstrated that lower strain rates promote SIMT due to the reduced effect of adiabatic heating. Additionally, it has been also observed that there is an increasing tendency of SIMT under low-temperature conditions (Egels et al. [15] and Kim et al. [16]). Furthermore, numerous studies have explored the influence of the loading direction and stress state on SIMT kinetics (Beese et al. [17] and Sohrabi et al. [18]). Table 1 compares SIMT kinetics under uniaxial and biaxial tensile loading conditions across various austenitic stainless steels, revealing that the stress-state dependence of SIMT kinetics varies depending on the material. LNG cargo tank membrane barriers are subjected to various complex loading conditions; however, the primary stress states are uniaxial and biaxial tensile loading due to thermal contraction in cryogenic environments. Therefore, a systematic investigation of SIMT behavior under these loading conditions is essential for evaluating the mechanical integrity of the membrane and ensuring its long-term structural reliability. Various methods have been proposed for measuring the volume fraction of transformed martensite. One approach, based on crystallography, involves electron backscatter diffraction (EBSD), which enables direct scanning and observation of the microstructure. However, since EBSD captures only a local region, results may vary depending on the scanned area, making it difficult to obtain statistically meaningful quantitative data for macroscale specimens or components. Another widely used crystallographic method is X-ray diffraction (XRD) measurement. Given that crystal structures and lattice planes produce characteristic diffraction peaks at specific angles, the integrated intensity of these peaks can be analyzed to quantitatively estimate the martensite volume fraction. Compared to EBSD, XRD allows for the analysis of larger sample areas, providing more statistically robust results. An alternative approach leverages the magnetic properties of the material. While austenite is non-magnetic, martensite exhibits ferromagnetic behavior, enabling the quantification of the martensite fraction using magnetic measurements. However, since the magnetic properties of martensite vary depending on chemical composition and manufacturing processes, a calibration process based on other measurement techniques is essential. Compared to the previous two methods, this approach is highly portable, non-destructive, and well-suited for industrial applications.

**Table 1:** Comparison of SIMT kinetics under UT and BT in austenitic stainless steel.

Material	SIMT Kinetics	References
SUS 304	Biaxial tension > uniaxial tension	[21,22]
SUS 301LN	Biaxial tension ≈ uniaxial tension	[23]
	Biaxial tension < uniaxial tension	[24]
SUS 201	Biaxial tension < uniaxial tension	[25]

In this study, a novel non-destructive inspection methodology utilizing the SIMT phenomenon in SUS304L is proposed to enable the early detection and quantitative assessment of damage in CCS membranes of LNG carriers. This method employs the ferromagnetic properties of transformed martensite, using a portable Feritscope to measure the ferrite number (FN) and indirectly evaluate the plastic deformation history and damage state. The feasibility and applicability of the proposed methodology were validated through material-scale experiments, and three complementary approaches are suggested for practical application in LNG carrier membrane inspections. First, outlier detection exploits the periodic structural characteristics of the membrane by comparing FN values measured at equivalent locations and using statistical techniques to identify regions with abnormally high FN values, enabling the early detection of potential defect sites. Second, quantitative damaged area analysis involves detailed FN measurements around suspected defect regions identified through outlier detection. This approach provides a quantitative assessment of the damage extent, offering objective data to determine the optimal repair scope. Third, time-series analysis measures FN values at key locations during regular inspections, analyzing the sequential data to track cumulative damage progression. This enables damage propagation rate prediction, structural residual life assessment, and the establishment of a risk-based maintenance strategy.

## 2. Materials and Methods

### 2.1. Material

**Figure 3:**
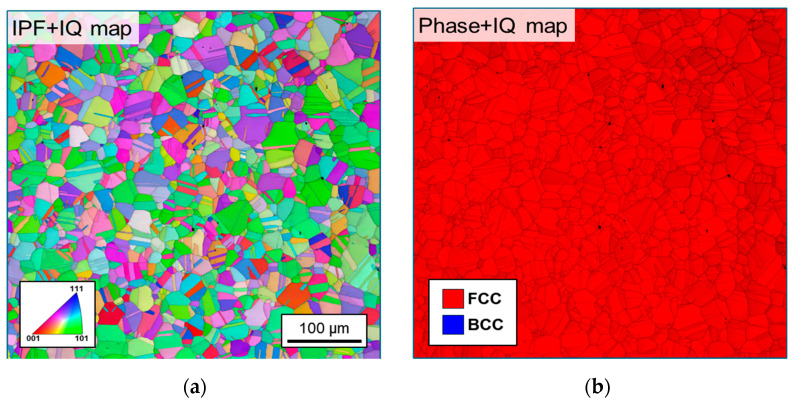
Microstructure of SUS304L: (**a**) inverse pole figure (IPF) along normal direction + image quality (IQ) map; (**b**) phase map.

This study investigates austenitic stainless steel 304L (SUS304L), which is widely used in LNG cargo containment systems (CCSs), using specimens with a thickness of 1.2 mm. The chemical composition of the SUS304L, analyzed in accordance with the ASTM A751-21 standard [26], is presented in Table 2. While SUS304L typically contains 8–10.5 wt.% nickel, Ryoo et al. [13] reported that an increase in Ni content reduces the kinetics of SIMT. In this study, SUS304L with a Ni content of 9.2 wt.% is investigated. Figure 3 illustrates the microstructure of as-received SUS304L, measured via electron backscatter diffraction (EBSD). Figure 3a shows an inverse pole figure (IPF) map along the normal direction (ND) overlaid with an image quality (IQ) map, and Figure 3b presents a phase map superimposed on an IQ map. The as-received microstructure consists entirely of the face-centered cubic (fcc) phase, with an average grain size of 19.2 µm and a standard deviation of 8.8 µm. Additionally, the microstructure contains several annealing twins.

### 2.2. Mechanical Test

**Figure 4:**
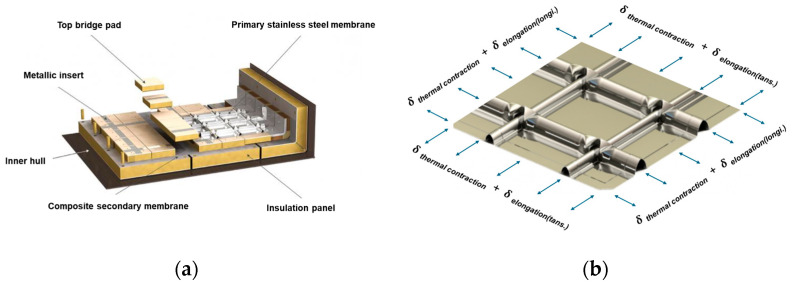
Schematic diagram: (**a**) Mark III insulation system components; (**b**) loading conditions on the primary membrane, including thermal contraction and hull elongation effects.

LNG cargo tank membranes are subjected to a complex interplay of loads during operation. These include hydrostatic and sloshing pressures, which vary depending on their position within the cargo hold, thermal contraction stresses arising from the cryogenic environment (−163 °C), and hull deformation loads resulting from phenomena such as hogging and sagging. Among these factors, thermal contraction due to cryogenic temperatures is the most dominant, generally subjecting the membrane to a tensile stress state (Figure 4). Previous studies have demonstrated that the stress state influences the kinetics of SIMT [14,24]. This finding underscores the necessity of considering loading conditions when predicting the SIMT behavior of structures exposed to complex mechanical environments, such as LNG cargo tank membranes, and highlights the critical role of loading conditions in structural integrity assessment.

To characterize the SIMT behavior of SUS304L under these complex loading conditions and to establish a foundation for a quantitative non-destructive evaluation system, this study conducted uniaxial tension (UT) and equi-biaxial tension (EBT) tests, which represent the most dominant loading conditions acting on membrane barriers.

All experiments were performed at room temperature under quasi-static conditions with a strain rate of 0.001 s^−1^, ensuring that adiabatic heating effects from plastic deformation and phase transformation were negligible [14,27]. Furthermore, to ensure the reproducibility of the experimental results, each test condition was repeated at least three times.

#### 2.2.1. Uniaxial Tensile Test

**Figure 5:**
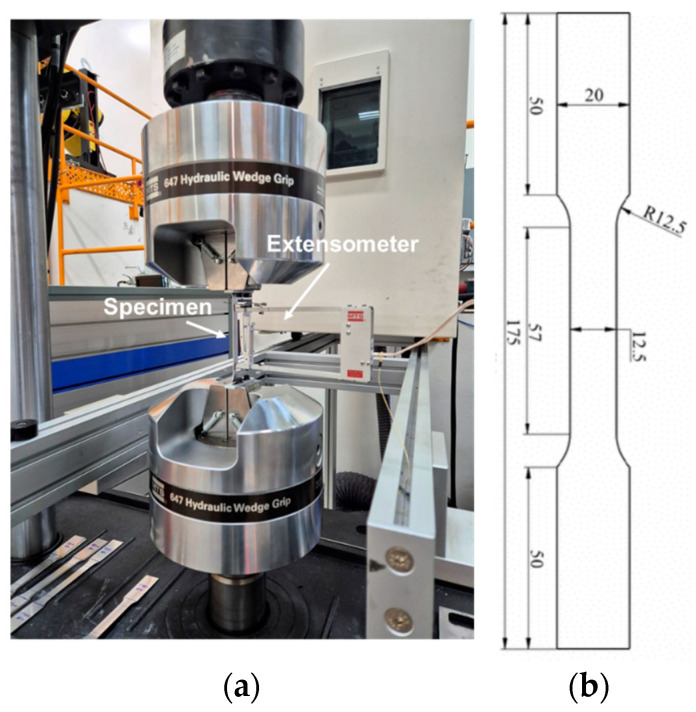
(**a**) Automatic universal testing machine and (**b**) specimen geometry and dimensions for UT test (unit: mm).

UT specimens were fabricated in accordance with the ASTM E8 standard [28] along the rolling direction (RD), transverse direction (TD), and diagonal direction (DD). The prepared specimens were precisely affixed within the designated grip regions of an MTS universal testing machine (UTM) (Figure 5). An integrated data acquisition system continuously recorded the applied load and corresponding displacement throughout the loading process. To ensure accurate measurement of material deformation, extensometers were symmetrically mounted on both sides of the gauge length to capture the total elongation within the gauge length. Additionally, to analyze microstructural evolution resulting from SIMT, interrupted tests were conducted at specific strain levels.

#### 2.2.2. Hydraulic Bulge Test

**Figure 6:**
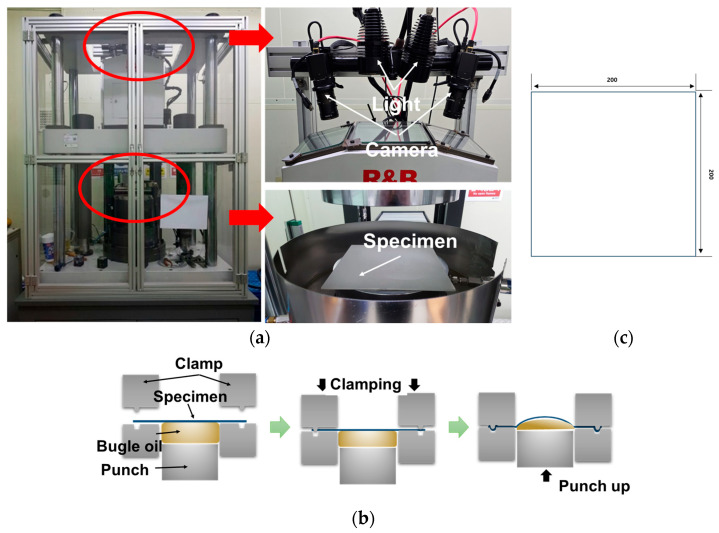
(**a**) Experimental setup for the hydraulic bulge test using the DIC system; (**b**) schematic illustration of the bulge test process; and (**c**) specimen geometry and dimensions for the bulge test (unit: mm).

The hydraulic bulge test was conducted to simulate EBT loading conditions. As shown in Figure 6, the specimens were fabricated in a square shape with dimensions of 200 mm × 200 mm. The experiments were performed using a universal sheet metal formability testing machine from R&B Co. (Daejeon, Republic of Korea), with key specifications listed in Table 3. The forming dome diameter (bulge diameter) was 100 mm, and the experimental setup and procedure are illustrated in Figure 6.

Bulge height measurement was conducted using a linear variable differential transformer (LVDT), while strain measurements were performed using a stereo-digital image correlation (DIC) system equipped with two charge-coupled device (CCD) cameras for three-dimensional deformation analysis. Camera calibration was performed to accurately reconstruct the 3D dome shape. The data obtained from each experiment were converted into stress–strain curves using membrane theory and were cross-validated with the strain field measured via DIC. Additionally, to compare the SIMT kinetics, interrupted tests were conducted at strain intervals similar to those used in UT tests, based on the measured strain.

During the hydraulic bulge test, the central region of the specimen experiences an equi-biaxial stress state, where the major stress ($\sigma_{1}$) and minor stress ($\sigma_{2}$) are equal ($\sigma_{b}$), expressed as

(1) $$\sigma_{1}{=\sigma }_{2}=\sigma_{b}$$

The equi-biaxial true stress ($\sigma_{b}$) relates the fluid pressure (p) applied to the specimen, the average radius of curvature (R), and the true thickness (t) of the specimen, calculated by

(2) $$\sigma_{b}=\frac{pR}{2t}$$

The radius of curvature is the reciprocal of the curvature, k, expressed as

(3) $$R=\frac{1}{k}\;\;\;\;,\;\;\;\;k=\frac{1}{2}{(k}_{xx}+k_{yy})$$

where k is obtained from the average of $k_{xx}\;$ along the x-direction and $k_{yy}$ along the y-direction. These equations are derived under the assumption that the specimen behaves similarly to a thin-walled spherical pressure vessel. Assuming plastic incompressibility and neglecting elastic deformation, the true thickness can be calculated using Equation (4), which relates the initial thickness ($t_{0}$) and the true thickness strain ($\varepsilon_{t}$):

(4) $$t=t_{0}\exp\left(\varepsilon_{t}\right)$$

Assuming plastic incompressibility, the total true thickness strain ($\varepsilon_{3}$) can be approximated using the major and minor true strains ($\varepsilon_{1}$ and $\varepsilon_{2}$, respectively) as given by

(5) $$\varepsilon_{3}≅-\varepsilon_{1}-\varepsilon_{2}$$

### 2.3. Microstructural Analysis

In this study, SIMT was analyzed through microstructural measurements and magnetic permeability testing to quantitatively establish the relationships among strain, transformed martensite volume fraction, and magnetic permeability. Initially, phase identification and quantification were performed via XRD analysis, followed by magnetic permeability measurements for the non-destructive evaluation (NDE) of martensitic transformation. Subsequently, EBSD analysis was conducted to investigate phase transformation from a crystallographic perspective. Through this systematic microstructural investigation, SIMT mechanisms under various loading conditions were elucidated, and the deformation state of the material was assessed using Feritscope measurements.

#### 2.3.1. Magnetic Permeability Measurement

The Feritscope operates by generating a low-frequency alternating magnetic field, which interacts with the specimen. A coil wound around the probe detects variations in the surrounding magnetic field caused by the magnetic constituents within the specimen. These variations induce a voltage in the measuring coil, which is proportional to the volume fraction of the magnetic content present in the specimen. For metastable austenitic stainless steel, since both γ-austenite and ε-martensite exhibit paramagnetic behavior, the resulting electrical signal exclusively reflects the contribution of the ferromagnetic α′-martensite. This measurement technique offers the advantages of being non-destructive and enabling rapid data acquisition. In this study, measurements were conducted within the range of 0.1–110 FN using the Feritscope FMP30 (Helmut Fischer GmbH, Sindelfingen, Germany).

#### 2.3.2. X-Ray Diffraction Measurement

XRD analysis was conducted to quantitatively determine the transformed martensite volume fraction as a function of strain. Specimens for XRD analysis were extracted from the gauge region of the uniaxial tensile test specimens and the peak height region of the hydraulic bulge test specimens. To minimize noise and ensure precise measurements, surface preparation was performed prior to analysis. Surface preparation commenced with mechanical polishing using SiC paper of various grit sizes. Subsequently, electropolishing was performed in a 10% perchloric acid solution to eliminate residual surface stress induced by mechanical polishing and to prevent any unintended phase transformation. XRD analysis was performed using a Malvern Panalytical X’Pert3 MRD X-ray diffractometer with CuKα radiation (Worcestershire, UK). The measurements were conducted under an accelerating voltage of 30 kV, within a 2θ range of 40–100°, and at a scan speed of 5°/min. After measuring the intensities of the austenite and martensite diffraction peaks, the martensite volume fraction was calculated using the Bentley–Smith method (Bentley et al. [29]):

(6) $$V_{\alpha^{\prime }}\left(\%\right)=\frac{\sum_{i=1}^{N_{\alpha }}\frac{I_{i}}{R_{i}}}{\sum_{i=1}^{N_{\gamma }+N_{\alpha }}\frac{I_{i}}{R_{i}}}\times 100$$

where *I* represents the integrated intensity of the diffraction peak and *R* denotes the material scattering factor, the values of which are provided by Bentley et al. [29]. The calculation utilizes the integrated intensities of the (110)α, (200)α, (211)α, and (220)α peaks for α’-martensite, as well as the (111)γ, (200)γ, (220)γ, (311)γ, and (222)γ peaks for austenite.

#### 2.3.3. EBSD Measurement

EBSD analysis was conducted to provide an in-depth assessment of microstructural evolution from a crystallographic perspective. To ensure measurement consistency, the same regions analyzed in the XRD measurements were examined. Mechanical polishing and electropolishing were performed identically to the preparation of the XRD specimens. Additionally, vibratory polishing was employed, using a colloidal silica suspension for an extended period to minimize surface deformation and ensure a high-quality surface suitable for grain boundary and crystallographic orientation analysis. EBSD data were acquired using the AZtec software (Version 4.2) package from Oxford Instruments and a suitable detector. The detector facilitated the acquisition of high-quality Kikuchi patterns, enabling quantitative evaluation of microstructural evolution through grain orientation analysis, phase analysis, and grain boundary analysis. Grain boundary analysis provided insights into deformation-induced grain refinement and texture development. A high spatial resolution is indispensable for the precise characterization of deformed microstructures. Table 4 summarizes the primary parameters employed for EBSD analysis in this study.

## 3. Results

### 3.1. Mechanical Property

**Figure 7:**
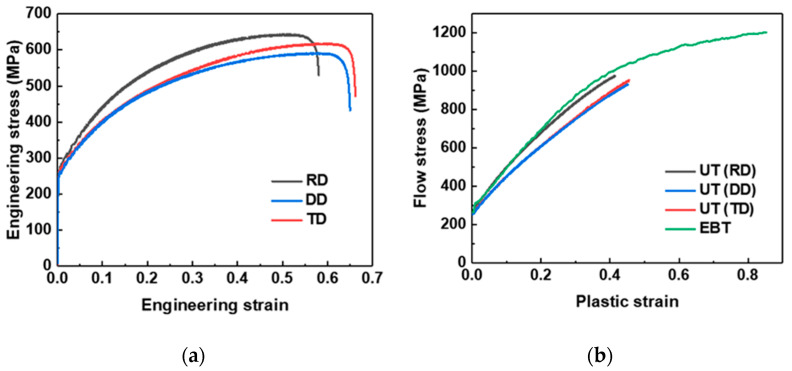
(**a**) Engineering stress–strain curves for UT tests conducted along three sheet orientations: rolling direction (RD), diagonal direction (DD), and transverse direction (TD); (**b**) comparison of flow stress curves for UT and EBT.

Figure 7a presents the engineering stress–strain curves obtained from UT tests conducted along the rolling direction (RD), diagonal direction (DD), and transverse direction (TD). The corresponding mechanical properties for each direction are summarized in Table 5. Slight variations in ultimate tensile strength and elongation were observed among the different loading directions. Furthermore, Figure 7b compares the flow stress curves between UT and EBT. All results exhibit pronounced work-hardening behavior, attributed to transformation strengthening induced by SIMT. The higher flow stress observed in EBT is likely a consequence of its enhanced SIMT kinetics, as discussed in the following section.

For the analysis of SIMT kinetics, the interrupted testing intervals in the UT test were determined based on uniform elongation and total elongation. Three distinct regimes were defined for the interrupted UT tests: (A) the pre-uniform elongation regime, with 10% engineering strain intervals, (B) the post-uniform elongation regime (necking region), and (C) the fracture point. The interrupted testing intervals in the EBT condition were set to correspond with those of the UT. Microstructural analysis, including XRD and EBSD, was conducted for Regime A in UT along RD and EBT, whereas Feritscope measurements were performed across all testing conditions to ensure a comprehensive assessment of SIMT evolution.

### 3.2. Quantitative Measurement of X-Ray Diffraction

**Figure 8:**
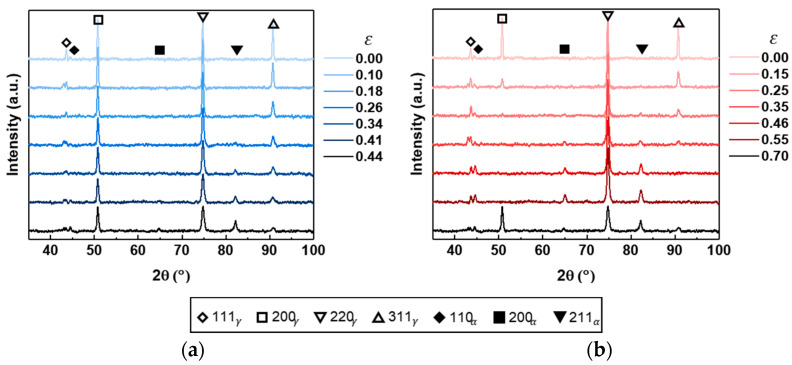
Evolution of XRD patterns of SUS304L with true strain under (**a**) UT (RD); (**b**) EBT.

Figure 8 shows the evolution of XRD patterns for the UT and EBT conditions as a function of true strain. In the XRD patterns, peaks corresponding to the austenite (γ) phase are identified at 2θ = 43.8°, 51.0°, 75.0°, and 91.0°, corresponding to the (111)γ, (200)γ, (220)γ, and (311)γ planes, respectively. Similarly, peaks corresponding to the martensite (α’) phase are observed at 2θ = 44.7°, 65.1°, and 82.4°, corresponding to the (110)α, (200)α, and (211)α planes, respectively. Under both loading conditions, as the strain increases, the intensity of the γ peaks gradually decreases, while the intensity of the α’ peaks progressively increases. Furthermore, the α’ peak intensities were higher under EBT compared to UT at similar strain levels, indicating that SIMT was more pronounced under EBT.

**Figure 9:**
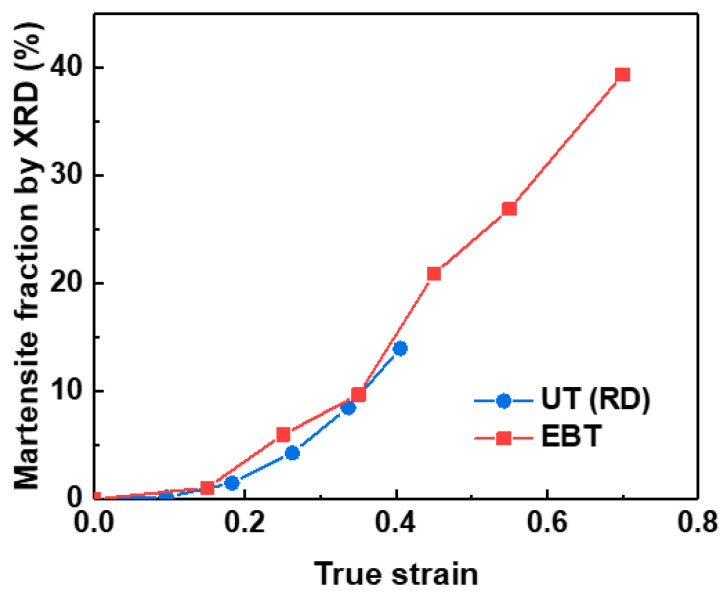
Comparison of the evolution of the transformed martensite volume fraction between UT and EBT, as calculated from XRD data.

Figure 9 quantitatively compares the evolution of the transformed martensite volume fraction for UT and EBT, as calculated using the Bentley–Smith method (Equation (6)) from XRD data. The results indicate that EBT exhibits higher SIMT kinetics than UT, aligning with trends observed in other experimental findings [21].

### 3.3. Quantitative Measurement of EBSD

**Figure 10:**
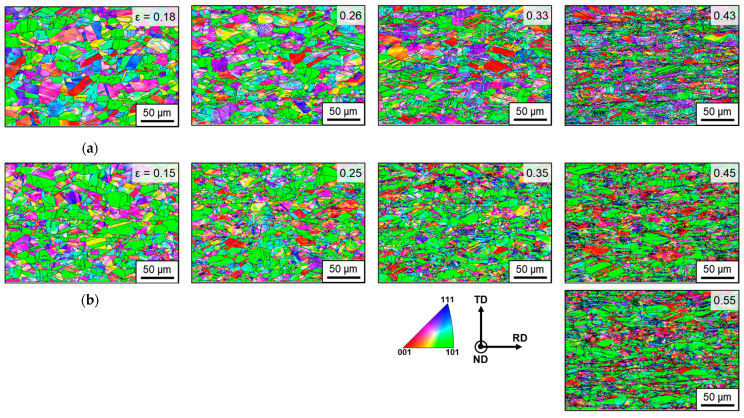
Evolution of IPF maps of SUS304L with true strain under (**a**) UT (RD); (**b**) EBT.

SIMT is governed by the interplay between the applied stress state and crystallographic orientation, observable at the microstructural level via EBSD analysis. This study employed EBSD analysis to investigate the correlation between SIMT and microstructural changes, including grain refinement and crystallographic textural evolution, under UT and EBT loading conditions. Figure 10 shows the IPF maps along the ND as a function of true strain. As deformation progresses, the initially coarse grains are refined due to the formation of subgrain boundaries. These boundaries form from accumulated dislocations during plastic deformation and eventually evolve into high-angle grain boundaries (HAGBs), leading to grain subdivision. This is a typical phenomenon in austenitic steels, where the dislocation density increases with strain. These dislocations rearrange to form stable low-angle grain boundaries (LAGBs) (subgrain boundaries), which subsequently develop into HAGBs through a process resembling dynamic recrystallization. A detailed analysis of grain structure and refinement is provided in Appendix A.

Table 6 shows the average grain size variation as a function of true strain under UT and EBT conditions. Under UT conditions, the FCC grain size steadily decreases from 12.2 ± 4.2 μm at a true strain of 0.18 to 6.8 ± 2.0 μm at a true strain of 0.43. Under EBT conditions, the decrease is less pronounced, from 10.1 ± 3.5 μm to 7.8 ± 2.1 μm. The BCC grains are significantly smaller than the FCC grains. Under UT conditions, the BCC grain size ranges from 1.8 ± 0.5 μm to 2.1 ± 0.6 μm and does not change significantly with strain. Under EBT conditions, the BCC grains are larger (4.5 ± 1.2 μm to 5.0 ± 1.1 μm) and show some fluctuations with strain. This suggests that martensite forms with a very small size within the austenite grains. According to the model proposed by Olson et al. [30], martensitic transformation preferentially occurs in micro-deformation structures, such as shear band intersections within austenite, and proceeds mainly via continuous nucleation of new martensite variants rather than the growth of existing martensite grains.

**Figure 11:**
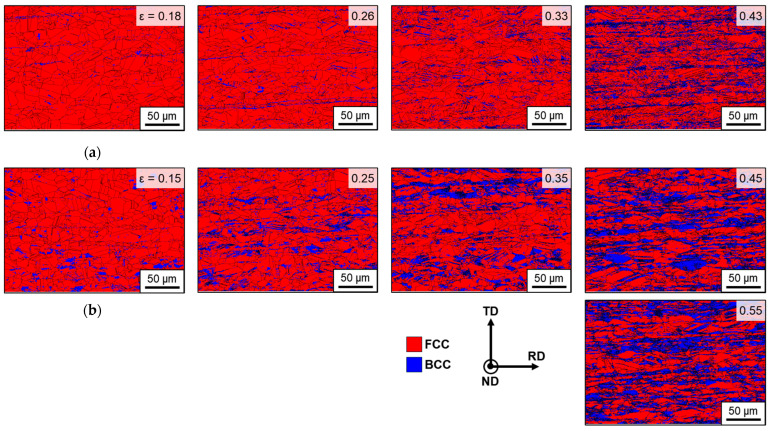
Evolution of phase maps as a function of strain for SUS304L under (**a**) UT; (**b**) EBT.

Figure 11 shows the evolution of phase maps under UT and EBT conditions as a function of strain, and Table 7 presents the corresponding quantitative phase fractions. The fraction of the BCC phase increases progressively with deformation under both loading conditions. When comparing equivalent strain levels, UT consistently exhibits a lower martensite volume fraction than EBT. In the UT condition (Figure 11a), significant grain refinement occurs near the uniform elongation (ε ≈ 0.43), accompanied by a rapid increase in martensite formation. In the UT conditions, martensite tends to form as thin, elongated laths along grain boundaries or within deformation bands. This phenomenon is attributed to increased strain heterogeneity within the microstructure, with heterogeneous regions, particularly shear bands, acting as nucleation sites for martensitic transformation [30]. In contrast, in the EBT condition (Figure 11b), martensite formation is more uniformly distributed from the early stages of deformation up to a true strain of 0.55. The morphology of martensite is more diverse under EBT conditions, with martensite also observed within the grains.

**Figure 12:**
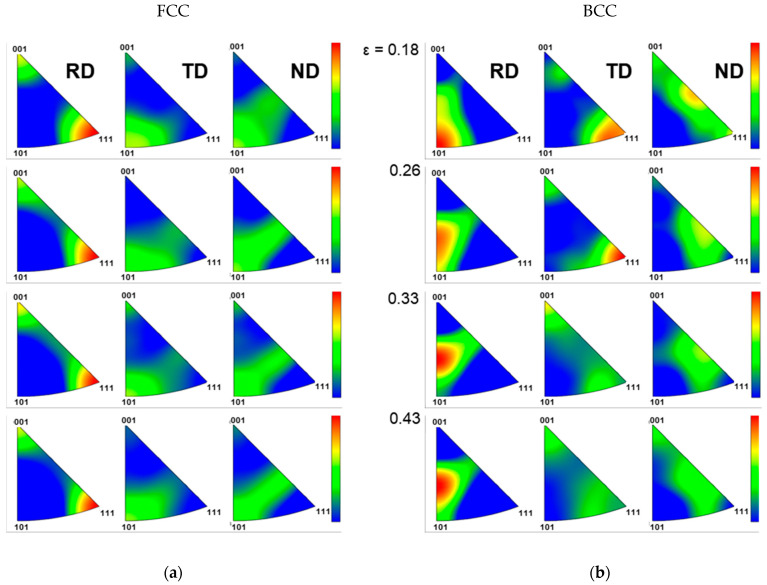
Crystallographic textural evolution (EBSD pole figures) of SUS304L with strain under uniaxial tension (UT): (**a**) FCC (austenite) phase; (**b**) BCC (α′-martensite) phase.

**Figure 13:**
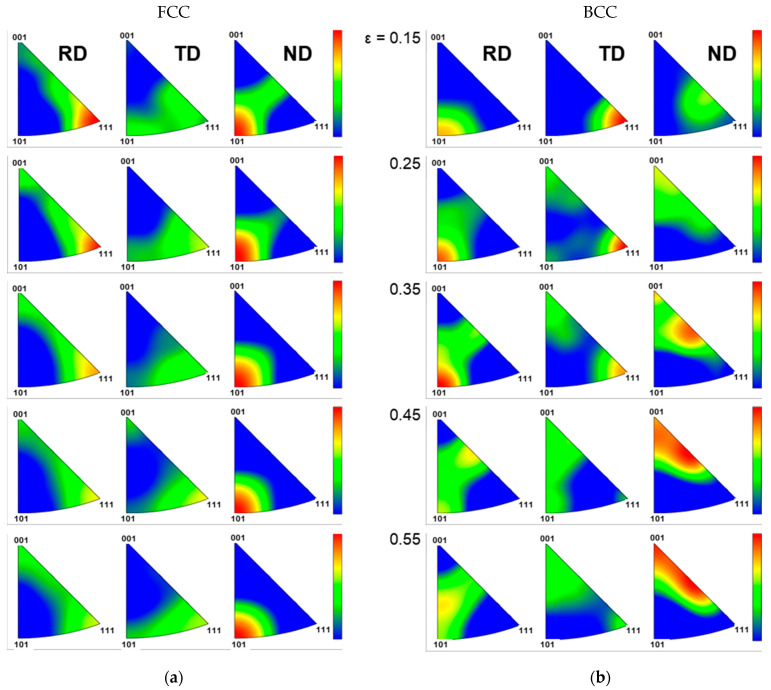
Crystallographic textural evolution (EBSD pole figures) of SUS304L with strain under equi-biaxial tension (EBT): (**a**) FCC (austenite) phase; (**b**) BCC (α′-martensite) phase.

Figure 12 and Figure 13 show the characteristics of crystallographic textural evolution under UT and EBT conditions, respectively, using pole figures. Under UT conditions (Figure 12), both the austenite and martensite phases initially exhibit relatively uniform orientation distributions along the RD, TD, and ND. However, as deformation progresses, the FCC phase develops a preferential <111> orientation along the RD due to the predominant activation of {111}<110> slip systems, while in the TD direction, concentration develops between <111> and <101>, and in the ND direction, concentration evolves between <101> and <001>. This textural evolution in the FCC phase influences the textural development of the BCC phase. This is a typical textural evolution trend observed in FCC metals during SIMT, and it is related to the Kurdjumov–Sachs (K-S) relationship, where the {111}_γ_ plane of austenite is parallel to the {110}_α′_ plane of martensite, and a <110>_γ_ direction within the {111}_γ_ plane is parallel to a <111>_α′_ direction within the {110}_α′_ plane. The BCC phase develops a strong <101> texture along the RD, and its intensity is comparable to or even stronger than that of the FCC <111> texture. In contrast, under EBT conditions (Figure 13), the activation of slip systems in both RD and TD directions means the FCC phase maintains more distributed orientation patterns. For the FCC phase, the intensity between <101> and <001> increases along the RD, and between <111> and <101> along the TD, while the <111> orientation intensity along the ND direction increases with increasing strain. The BCC phase also shows significant textural evolution, particularly along the ND, where the intensity between <001> and <101> increases notably with increasing strain. While texture changes in the FCC phase are less pronounced under EBT compared to UT conditions, the BCC phase shows a more significant textural evolution, especially in the higher strain ranges (ε > 0.35).

The final zero solution values in the EBSD data were approximately 3.0% for UT and 10.8% for EBT (Table 7), indicating that the complex deformation state induced by multi-directional stresses made EBSD pattern indexing more challenging. This implies that local misorientations and defect densities within the grains were significantly higher under EBT conditions. In summary, the XRD and EBSD analyses clearly demonstrate that the strain-induced martensitic transformation (SIMT) in SUS304L stainless steel is significantly influenced by the loading condition, with SIMT being promoted under EBT conditions due to the activation of multiple slip systems. This is supported by the observed more dispersed crystallographic texture (Figure 13) and higher martensite volume fraction (Figure 11, Table 7). Furthermore, the direct γ → α’ transformation without evidence of an intermediate ε-martensite phase, as confirmed by XRD analysis (Figure 8), and the comprehensive EBSD data, combined with previous research [25], suggest that in the UT conditions, deformation twinning acts as the primary deformation mechanism, suppressing α’-martensite transformation, whereas in the EBT conditions, the activation of multiple slip systems and the increased nucleation sites due to their intersections result in a higher α’-martensite fraction.

### 3.4. Quantitative Measurement of Feritscope

**Figure 14:**
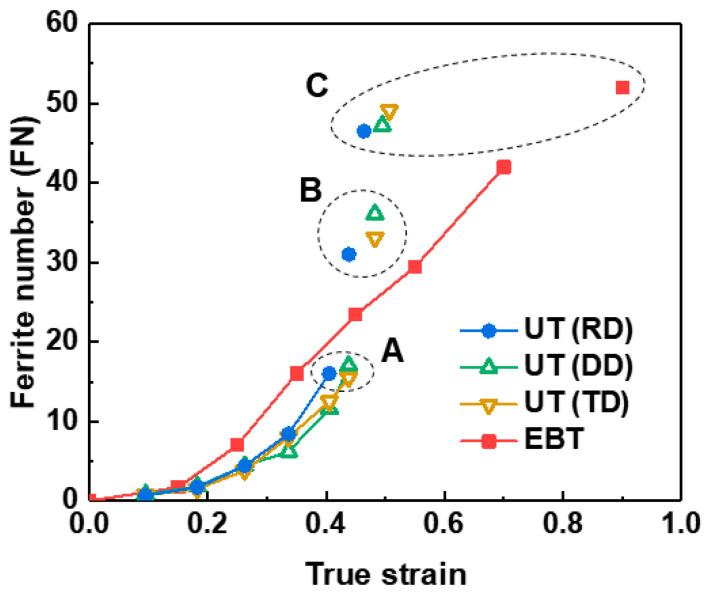
Evolution of FN with true strain under UT and EBT.

Figure 14 presents the evolution of the ferrite number (FN) with respect to true strain in the UT and EBT. In both conditions, FN increases with increasing strain due to SIMT, with a more pronounced increase observed under EBT. This suggests that FN measurements can be used to estimate the material’s deformation history. Notably, since SUS304L, an austenitic stainless steel, exhibits no FN value without plastic deformation, the detection of an FN value indicates plastic strain, signifying a potentially critical state for structural integrity. Therefore, FN measurement offers the potential for early detection of structural defects or anomalies in membrane-type cargo tanks constructed from SUS304L. In this study, Feritscope measurements were conducted to characterize UT specimens in the TD and DD directions, as well as fractured specimens. Conventional techniques such as XRD and EBSD are inherently limited in these cases.

The evolution of FN can be categorized into three distinct regimes: (A) pre-uniform elongation (UEL), (B) post-UEL (necking region), and (C) fracture. In regime A, the FN increases gradually across all specimens, indicating a progressive and relatively homogeneous martensitic transformation under uniform deformation conditions. While a difference in FN values between UT and EBT is observed, it is not substantial. As deformation progresses into regime B, a sharp escalation in FN is observed in UT specimens due to intensified strain localization, which accelerates SIMT. In contrast, under EBT conditions, with more uniformly distributed strain, the increase in FN remains comparatively moderate. This discrepancy underscores the challenge of accurately assessing the strain state of structural components subjected to unknown loading conditions based solely on FN measurements. Finally, in regime C, corresponding to the fracture point, FN values across all specimens converge to approximately 50, regardless of the loading mode. This suggests a critical threshold of the transformed martensite fraction associated with fracture in this material.

### 3.5. XRD-Based Calibration of the Feritscope

While the Feritscope was originally developed to quantify the δ-ferrite content in austenitic stainless steels, it can be adapted to measure the volume fraction of α’-martensite. However, variations in magnetic permeability due to the extent of deformation, microstructure, and alloy composition necessitate systematic calibration for quantitative analysis [24,31,32,33,34,35,36,37,38]. Talonen et al. [39] conducted a comparative study of various calibration techniques, including XRD, Satmagan, and density-based methods, for SUS301LN and SUS304 subjected to tensile deformation and cold rolling. Their findings suggested that calibration using the Satmagan method provided the highest accuracy, and the relationship between FN and the volume fraction is expressed as follows:

(7) $$V_{\alpha^{\prime }}\left(\%\right)=Z\times FN\;(where\;Z=1.7\;with\;R^{2}=0.987)$$

However, even within the study by Talonen et al. [39], the XRD-based calibration factor showed significant variations depending on the specimen processing history and measurement conditions, as follows.

Case 1: Cold rolled material, Cr Kα radiation:

(8) $$V_{\alpha^{\prime }}\left(\%\right)=Z\times FN+S\;(\mathrm{where}\;Z=2.676,\;S=1.203\;\mathrm{with}\;R^{2}=0.973)$$

Case 2: Cold rolled material, Co Kα radiation:

(9) $$V_{\alpha^{\prime }}\left(\%\right)=Z\times FN+S\;(\mathrm{where}\;Z=1.845,\;S=4.140\;\mathrm{with}\;R^{2}=0.992)$$

Case 3: Tensile strained material, Cr Kα radiation:

(10) $$V_{\alpha^{\prime }}\left(\%\right)=Z\times FN+S\;(\mathrm{where}\;Z=1.422,\;S=0.696\;\mathrm{with}\;R^{2}=0.961)$$

The findings of Talonen et al. [39] demonstrate that even within the same study, subtle variations in XRD measurement conditions, such as the type of X-ray source and the diffraction peaks selected for analysis, can significantly influence the XRD-based calibration factor. This underscores the sensitivity of XRD-based calibration not only to measurement parameters but also to the specimen’s texture and microstructure. Despite the recognized accuracy of the Satmagan method, this study adopted XRD-based Feritscope calibration. While Satmagan provides only the volume fraction of α’-martensite, XRD analysis offers not only phase quantification but also comprehensive microstructural information, including crystallographic texture, lattice strain, and phase transformation mechanisms. This comprehensive information is crucial for a deeper understanding of the complex material behavior occurring under diverse loading conditions. Furthermore, while acknowledging the precision of Satmagan for absolute quantification, this study prioritizes evaluating the consistency of Feritscope measurements in the UT and EBT conditions, rather than determining the absolute α’-martensite content. If the Feritscope exhibits predictable behavior across these two loading conditions, XRD-based calibration, coupled with microstructural information, is considered more suitable for subsequent applications, such as health assessment and remaining life prediction of real structures, including LNG cargo tanks. In essence, this study focuses on evaluating the reliability of the correlation between Feritscope measurements and α’-martensite content, and the consistency of Feritscope measurements in the UT and EBT conditions is crucial for validating the proposed non-destructive evaluation technique. Therefore, instead of pursuing a highly precise calibration factor for absolute quantification, the primary objective was to determine whether the Feritscope readings qualitatively reflect the α’-martensite transformation trend, even under different loading conditions. In particular, XRD provides information near the surface, making it useful for evaluating structures such as LNG cargo tank membranes, where surface deformation and phase transformation are important.

**Figure 15:**
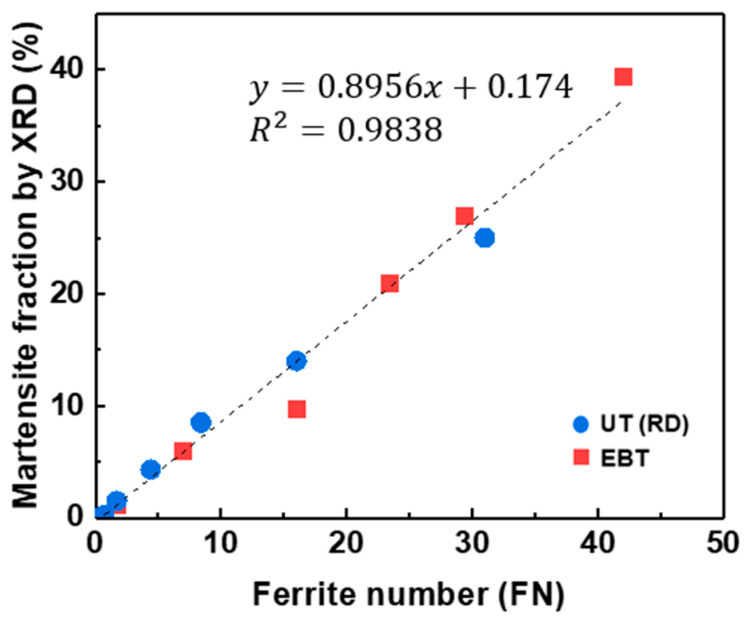
Correlation between α’-martensite volume fraction measured by XRD and Feritscope readings.

In that context, this study derived the following linear relationship between the Feritscope readings and the α’-martensite volume fraction measured by XRD (Equation (11) and Figure 15):

(11) $$V_{\alpha^{\prime }}\left(\%\right)=Z\times FN+S\;(where\;Z=0.895,S=0.174\;with\;R^{2}=0.983)$$

The high R^2^ value indicates a strong linear correlation between the Feritscope readings and the α’-martensite volume fraction measured by XRD, demonstrating the utility of the Feritscope for comparing the relative trends of SIMT under various loading conditions.

## 4. Discussion

### 4.1. Proposal of Inspection Methodology Based on Magnetic Permeability

Based on the material-level experiments in Section 3.5, which established a quantitative correlation between the FN increase due to SIMT and material damage, this section explores the practical application of Feritscope measurements for structural health monitoring of LNG carrier CCSs. The plastic deformation of SUS304L induces a γ-to-α’ transformation, which can be quantified by measuring the FN using a Feritscope. This finding is significant because it demonstrates the potential of Feritscope-based non-destructive testing to indirectly assess the plastic deformation history of a structure and, therefore, the degree of structural damage. Given its non-destructive nature and high portability, the Feritscope can serve as an effective tool for in situ inspections, such as routine assessments of LNG carrier CCSs.

The material-level experimental results suggest that, with a comprehensive understanding of the relationship between FN values and failure established through extensive experiments under various temperature and loading conditions, FN measurements could potentially be utilized to predict structural failure. However, establishing a direct and generalized relationship between FN values and failure, while simultaneously accounting for the complex and diverse loading histories experienced by the membrane barrier of an operating LNG carrier and the various environmental factors (temperature fluctuations, seawater exposure), is an exceptionally challenging task. It requires vast experimental datasets and long-term field validation.

To overcome these limitations and effectively leverage the potential of FN measurements identified in material-level experiments for the actual inspection of LNG carriers, this study proposes a novel inspection methodology based on relative, rather than absolute, FN values. This approach integrates the following three key strategies, taking into account the characteristics of the LNG carrier membrane barrier:

- Outlier detection: This method identifies regions exhibiting abnormally high FN values by conducting a relative comparison within periodic structural patterns subjected to similar loading conditions.
- Quantitative damaged area analysis: This approach quantitatively assesses the extent of damage in a suspected area by comparing its FN values with those of the surrounding regions, thereby aiding in determining the optimal repair scope.
- Time-series analysis: By performing regular Feritscope measurements, this method monitors cumulative damage progression over time, enabling predictive assessments of the structure’s remaining service life.

These approaches are designed to be complementary, thereby enhancing the reliability and efficiency of the inspection process.

#### 4.1.1. Outlier Detection

The experimental results in Section 3.4 showed that the FN values of the fractured specimens tended to converge to approximately 50 (C regime in Figure 14). This suggests that Feritscope measurement can be used to identify areas nearing failure. However, to ensure the structural integrity of the CCS and to detect potential defects at an early stage, it is crucial to analyze FN values and detect damage in the pre-UEL regime (A regime in Figure 14). As shown in Figure 14, the FN values in the pre-UEL regime vary depending on the loading conditions, indicating that the degree of SIMT can vary even at the same strain level depending on the stress state. Additionally, factors such as specimen curvature, specimen thickness, cladding thickness, edge distance, and surface roughness, which influence Feritscope measurements, need to be considered. These factors make it difficult to assess the degree of damage in a structure using the FN value as an absolute criterion.

**Figure 16:**
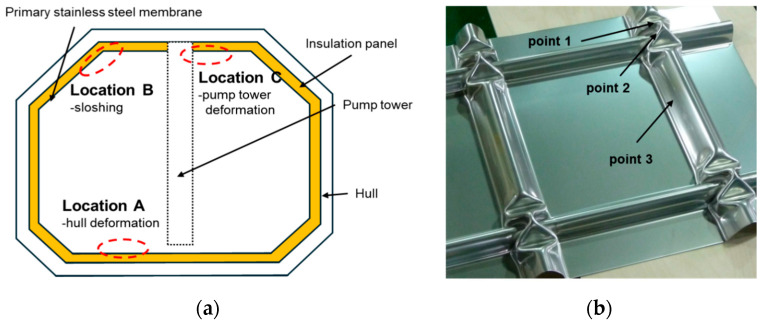
(**a**) Schematic diagram of an LNG carrier CCS, with typical loading zones indicated for each wall; (**b**) typical check points for FN measurement on the membrane.

To address these issues and enhance the effectiveness of Feritscope-based inspection, this study introduces the concept of outlier detection. The CCS membrane of an LNG carrier has a structural characteristic in which identical shapes subjected to similar loads are repeated, as shown in Figure 16a. Leveraging this characteristic, it is possible to compare the FN values measured at the same location on each membrane structure and identify outliers with abnormally high FN values using statistical techniques. For instance, in medical imaging, a normal range is established based on a large amount of data from normal tissues, and cases that deviate from this range are considered anomalies. In the case of the membrane barrier, the same concept can be applied by accumulating FN data from identical locations on regularly arranged membrane structures, establishing a normal range, and using this range to detect outliers during inspection.

**Figure 17:**
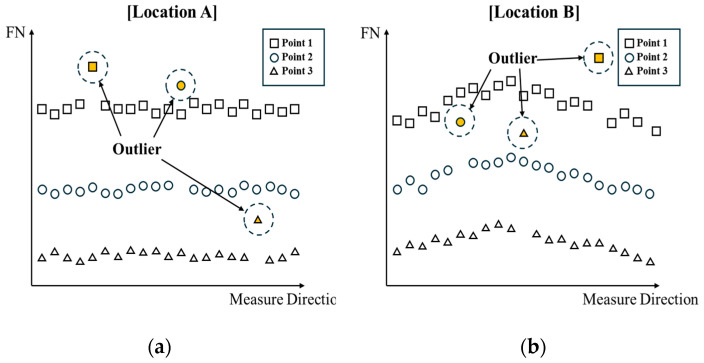
Example of longitudinal Feritscope measurement results showing potential outliers and different FN distributions for: (**a**) location A; (**b**) location B.

As shown in Figure 16a, in addition to the thermal contraction load generated in the cryogenic environment (−163 °C), the membrane barrier is exposed to various loading conditions depending on its location, such as pressure from sloshing, load from the pump tower loads, and hull deformation loads. These differences in loading conditions affect the SIMT behavior at each location, so the distribution of FN values may vary for each tank wall, as shown in Figure 17.

Figure 16 shows an example of selecting the locations where cracks or significant deformation (such as collapse-type failure) are likely to occur on the membrane as the main inspection points (check points). By measuring FN values at these main inspection points using a Feritscope and analyzing the distribution of FN values for each location, as shown in Figure 17, it is possible to identify membranes with statistically significant outliers. These outliers indicate that the membrane has experienced greater plastic deformation than other membranes or is at a higher risk of damage.

The data-driven approach proposed in this study has the advantage of offsetting the effects of membrane geometry, such as curvature, edges, and welds, which can affect Feritscope measurements. This is because it detects outliers by comparing relative values between membranes rather than absolute FN values. Furthermore, by continuously accumulating data on FN values under normal conditions and in cases of damage, it is possible to establish a critical FN threshold for determining the need for repair and to improve the reliability of inspection.

#### 4.1.2. Quantitative Damaged Area Analysis

Conventional visual inspection methods, widely used in practice, have inherent limitations in quantitatively assessing the impact of localized damage, such as significant deformation (like collapse-type failure) or cracking, on adjacent areas. This is a critical issue, as stress concentration and plastic deformation can develop in regions surrounding the damaged membrane, potentially compromising the structural integrity of the wider area. In Section 3.5, a quantitative relationship between strain and FN in SUS304L was established, implying that Feritscope measurements can be used to quantitatively assess the degree of plastic deformation and damage. As a validation step for applying the Feritscope measurement-based quantitative assessment to real structures, FN values were measured and analyzed across various regions of the hydraulic bulge specimens used in Section 3.

**Figure 18:**
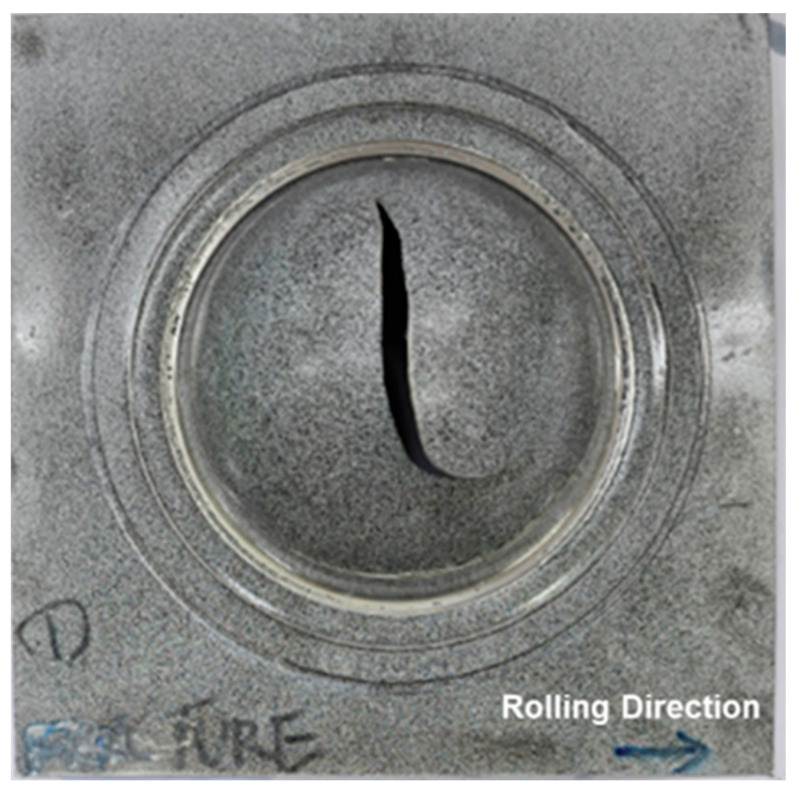
Fracture behavior in an SUS304L hydraulic bulge test.

Figure 18 shows a fractured SUS304L bulge test specimen. The fracture primarily occurred perpendicular to the RD, which is attributed to the material’s anisotropy, specifically the differences in elongation and R-value, as shown in Table 5. The lower uniform elongation of the RD (51.3%) compared to the TD (58%) indicates a greater resistance to deformation in the RD, and the higher R-value of the RD (0.90) compared to the TD (0.80) indicates that thickness-direction deformation occurs more easily than width-direction deformation in the RD. This anisotropy causes deformation to concentrate in the relatively weaker TD during the bulge test, ultimately leading to fracture perpendicular to the RD.

In material-level tests, as demonstrated in Figure 18, the loading direction and resulting fracture path (perpendicular to the RD) are relatively straightforward to predict due to the controlled, uniaxial nature of the loading conditions. However, in real-world structures like LNG carrier membranes, the loading conditions are far more complex, involving multiple, simultaneous loads with varying magnitudes and directions. This complexity makes it exceedingly difficult to predict deformation patterns and potential fracture locations and quantitatively assess the extent of damage using simple analytical approaches. To address this challenge, Feritscope-based FN measurements offer a crucial advantage.

**Figure 19:**
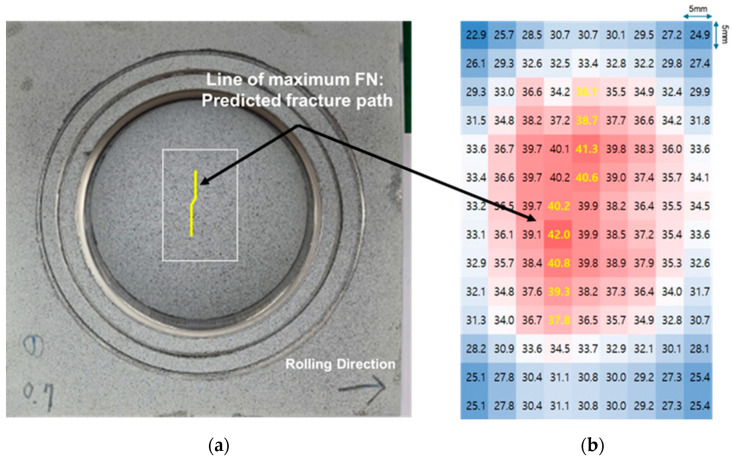
(**a**) Prediction of fracture path in SUS304L bulge test specimen (ε = 0.7) using FN measurements; (**b**) FN distribution map.

Figure 19a depicts the bulge test specimen at a true strain of 0.7. A key finding of this study is that Feritscope measurements, as illustrated in Figure 19b, enable quantitative assessment of the material’s strain state. Recall from Section 3.5 that an FN value of 50 corresponds to the fracture threshold for this material. Consequently, regions exhibiting FN values exceeding 40 in Figure 19b are identified as being at a high risk of imminent fracture. By connecting the loci of maximum FN values, a predicted fracture path is delineated, as illustrated in Figure 19a. This path represents the anticipated trajectory of crack propagation. Furthermore, the visualization of FN values, as in Figure 19b, facilitates a straightforward assessment of the damaged area within the structure.

The close resemblance between the actual fracture path in Figure 18 and the predicted fracture line based on FN measurements in Figure 19 suggests that FN measurements can be used for fracture prediction and, furthermore, for structural condition assessment in real structures. That is, although it is impossible to confirm visually, the degree of damage to the structure can be visualized and the risk of potential fracture can be detected in advance through FN measurements.

Therefore, if a high FN value is detected in a specific membrane through outlier detection, FN measurements should be performed not only on that membrane but also on the surrounding membranes to quantitatively determine the extent of the damaged area. By measuring FN values in the adjacent areas and comparing them with the baseline FN distribution (established through outlier detection), the extent of the affected area can be quantitatively determined, and this information can be used to determine the optimal repair scope.

This approach, based on Feritscope measurements, complements the qualitative limitations of visual inspection and enables a more accurate and reliable assessment of structural integrity. This contributes to enhancing the safety and maintenance planning of LNG carriers and can be utilized to improve the efficiency and accuracy of repairs by focusing on areas with quantifiable damage and to prevent potential future failures.

#### 4.1.3. Time-Series Analysis

The outlier detection and quantitative damaged area analysis methods, proposed in Section 4.1.1 and Section 4.1.2, respectively, are valuable for assessing the structural condition at a specific point in time. However, for structures subjected to repeated loading over extended periods, such as LNG carrier membranes, it is crucial to predict the cumulative damage progression over time. To that end, this study proposes a time-series analysis of FN measurement data obtained using a Feritscope.

By performing Feritscope measurements at key locations on the membrane barrier during regular inspections and analyzing the data chronologically, it is possible to track changes in FN at locations where structural stability may be compromised. This allows for a quantitative analysis of the cumulative damage progression in the structure and can be further utilized to predict the rate of damage propagation and assess the remaining service life of the structure.

**Figure 20:**
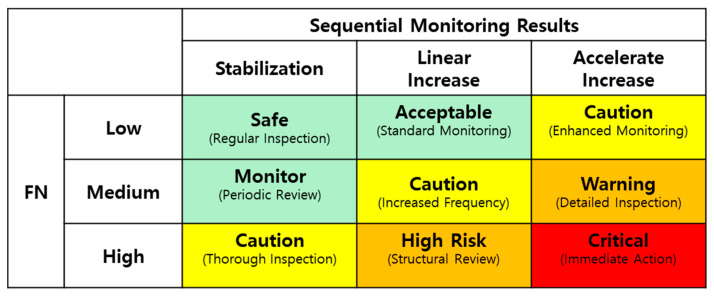
Risk assessment matrix based on sequential monitoring results of FN formation in membrane barriers.

The risk assessment matrix presented in Figure 20 systematizes this time-series analysis to evaluate the risk level associated with FN changes in membrane barrier structures. This assessment is based on two key parameters:

- Rate of FN increase: This parameter quantifies the increase in FN between consecutive inspection cycles and is categorized into three levels: Low, Mid, and High. It serves as an indicator of the current damage progression rate relative to previous inspection results. Initially, a relative criterion (e.g., percentage increase compared to the previous inspection) can be applied, and as data accumulates, more precise and quantitative threshold values (e.g., a specific FN increase) can be established.
- Temporal pattern of FN increase: This parameter classifies FN variation trends over multiple inspection cycles into three categories: stabilization, linear increase, and accelerated increase. It serves as an indicator of cumulative damage progression over time, capturing not only the instantaneous rate of FN increase but also the long-term trend, thereby enabling a more comprehensive assessment of the structural risk level.

The risk level for each inspection point can be evaluated by comprehensively considering these two parameters, leading to the determination of various inspection and maintenance strategies, as presented in Figure 20. These strategies range from regular inspection to more intensive actions: standard monitoring, enhanced monitoring, periodic review, increased frequency, and immediate intervention (e.g., thorough inspection and structural review). For example, a location with a high rate of FN increase and an accelerated increase temporal pattern would be classified as critical, requiring immediate action. Conversely, a location with a low rate of FN increase and a stabilization temporal pattern would be considered safe, requiring only regular inspection.

This time-series analysis-based risk assessment offers several advantages beyond simple anomaly detection: (1) the ability to predict the rate of damage progression within the structure, (2) enhanced predictive reliability through continuous data accumulation, and (3) improved objectivity and consistency in the interpretation of inspection results. Furthermore, by defining the FN values corresponding to low/mid/high, and the patterns corresponding to stabilization/linear increase/accelerated increase based on concrete data (FN measurement values, elapsed time, etc.), objectivity and consistency in the interpretation of inspection results can be ensured.

## 5. Conclusions

This study has introduced and experimentally validated a novel non-destructive inspection methodology for the cargo containment system membranes of LNG carriers, leveraging the SIMT phenomenon in austenitic stainless steel (SUS304L). The primary aim was to overcome the limitations of conventional inspection techniques, such as visual inspection and vacuum box testing, which are often subjective, limited in detecting incipient damage, and lacking in quantitative assessment capabilities. To address these issues, this study developed a highly reliable and quantitative structural health monitoring methodology based on Feritscope measurements.

UT and EBT were conducted on SUS304L specimens, accompanied by Feritscope measurements, XRD, and EBSD analyses to characterize the SIMT behavior and microstructural evolution. The experimental results demonstrated that FN values increased with increasing strain under both loading conditions, with a more pronounced SIMT observed under EBT. This is attributed to the more uniform distribution of martensite, higher martensite volume fraction, and enhanced nucleation due to the activation of multiple slip systems and shear band intersections, as evidenced by the EBSD analysis.

The evolution of FN values could be categorized into three distinct regimes: (A) a gradual increase in FN corresponded to the uniform deformation regime, (B) a sharp increase in FN was associated with the localized deformation (necking) regime, and (C) a convergence of FN values around 50 was observed in fractured specimens. These findings from the material-level tests confirmed the utility of FN measurements via the Feritscope for quantitatively assessing the deformation history and damage state of the membrane.

However, it is recognized that the complex loading history experienced by LNG carrier membranes in service poses challenges in generalizing a direct relationship between FN values and failure. Additionally, factors influencing Feritscope measurements, such as specimen geometry and surface roughness, need to be considered.

To address these limitations and enhance practical applicability, this study proposes three complementary inspection approaches based on FN measurements:

- Outlier Detection: This approach leverages the repetitive structural features of the membrane and uses statistical methods to identify regions with abnormally high FN values, thus enabling early detection of potential defect locations.
- Quantitative Damaged Area Analysis: This involves detailed FN measurements around a suspected defect area to quantitatively assess the extent of damage and guide repair scope decisions.
- Time-Series Analysis: This entails periodic FN measurements to track the cumulative damage progression over time, predict the remaining useful life, and inform risk-based maintenance strategies.

The methodology presented in this study introduces a data-driven, quantitative assessment to complement traditionally qualitative inspection methods, thereby contributing to the improved safety and maintenance efficiency of LNGC cargo containment system membranes. The time-series analysis-based risk assessment matrix, in particular, offers a practical decision-making tool for field applications and represents a novel approach for predicting the structural lifespan.

Moving forward, future research should focus on conducting additional experiments under cryogenic and cyclic loading conditions, evaluating the influence of welding and performing field validations on operational LNG carriers to further enhance the reliability and applicability of the proposed methodology. Moreover, integrating finite element analysis (FEA) can aid in predicting stress concentration areas, refining inspection point selection, and establishing refined criteria for the risk assessment matrix. This research is expected to contribute to advancements in structural health monitoring and maintenance technologies in the field of ocean engineering.

## Figures and Tables

**Table 2:** Chemical composition of SUS304L (wt.%).

Material	Chemical Composition							
SUS304L	Ni	C	Si	Mn	S	P	Cu	Cr
	9.2	0.016	0.62	1.2	0.003	0.014	0.11	18.1

**Table 3:** Conditions for the hydraulic bulge test.

Parameter	Value
Drawing force	80 tonF
Blank holding force	80 tonF
Drawing speed	0.02 mm/s
Bulge diameter	100 mm
Specimen thickness	1.2 mm
Strain measurement	ARAMIS DIC system

**Table 4:** EBSD scanning conditions.

Parameter	Value
Acceleration voltage	20 kV
Magnification	×500
Scan area	260 × 180 μm^2^
Step size	0.2 μm
Indexing rate	99.2%

**Table 5:** Mechanical properties of SUS304L in RD, DD, and TD.

	0.2% Yield Stress (MPa)	Ultimate Tensile Strength (MPa)	Uniform Elongation (%)	Total Elongation (%)	R-Value (ε = 15%)
RD	262	641	51.3	59	0.90
DD	253	593	57	64	1.26
TD	254	611	58	66	0.80

**Table 6:** Average grain size (μm) variation with strain under UT and EBT conditions.

True Strain	UT (FCC)	UT (BCC)	EBT (FCC)	EBT (BCC)
0.18/0.15	12.2 ± 4.2	1.8 ± 0.5	10.1 ± 3.5	4.5 ± 1.2
0.26/0.25	11.3 ± 3.7	2.1 ± 0.6	9.2 ± 2.9	3.5 ± 0.9
0.33/0.35	10.0 ± 3.1	1.9 ± 0.5	8.8 ± 2.6	4.2 ± 1.1
0.43/0.45	6.8 ± 2.0	2.1 ± 0.6	7.8 ± 2.1	5.0 ± 1.1
0.55	-	-	7.6 ± 1.9	3.9 ± 1.0

**Table 7:** Phase fractions (%) identified from EBSD as a function of true strain under UT and EBT conditions.

**UT** **(True Strain %)**	**0.18**	**0.26**	**0.33**	**0.43**
FCC	97.1	91.7	85.4	67.6
BCC	2.2	5.5	10.9	29.4
Zero solution	0.7	2.8	3.7	3.0
**EBT** **(True Strain %)**	**0.15**	**0.25**	**0.35**	**0.45**
FCC	91.1	79.7	70.4	54.8
BCC	8.1	17.4	24.9	34.4
Zero solution	0.8	2.9	4.7	10.8

**Figure A1:**
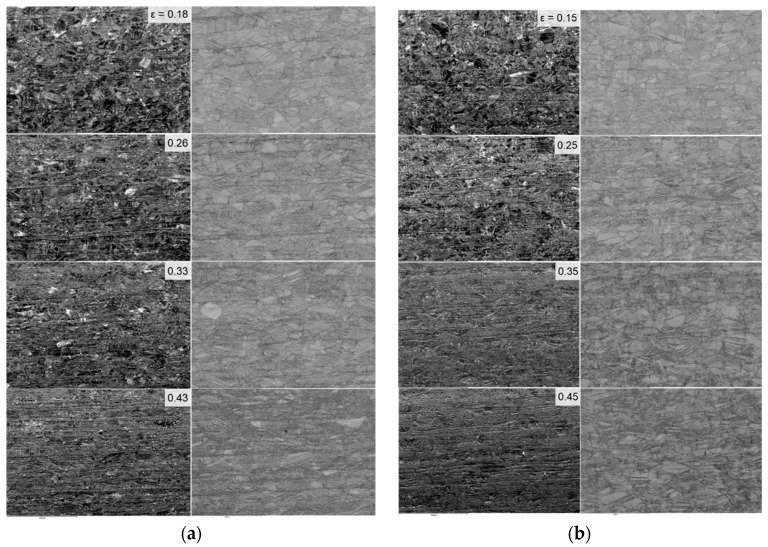
Microstructural evolution of SUS304L: FSD (**left**) and BC (**right**) images under (**a**) UT; (**b**) EBT conditions as a function of strain.

## Data Availability

The original contributions presented in this study are included in the article. Further inquiries can be directed to the corresponding authors.

## Appendix A

### Appendix A.1. Detailed EBSD Analysis: Grain Morphology and Refinement

Figure A1 presents the microstructural evolution under UT and EBT conditions as a function of true strain, using forward scatter detector (FSD) images (left) and band contrast (BC) images (right). The FSD images reveal subtle variations in strain within the grains, while the BC images clearly delineate the grain boundaries. Under UT conditions (Figure A1), as the true strain increases, the FSD images show an increasing number of lines and dark regions within the grains, indicating an increase in dislocation density, subgrain formation, and the development of micro-deformation bands. The BC images show that the grains become progressively finer with increasing strain, and the grain boundaries change in morphology. In particular, a significant grain refinement is observed near the uniform elongation (ε = 0.43), which is associated with the rapid increase in martensite formation discussed in conjunction with Figure 11a. Strain heterogeneity within the microstructure induced by deformation, especially regions such as shear bands, acts as nucleation sites for martensitic transformation [30]. Indeed, the role of shear band intersections in deformation-induced martensitic transformation (DIMT) kinetics has been specifically incorporated into recent crystal plasticity models to improve predictive capabilities for advanced high-strength steels like QP980 [40]. It has also been reported that shear band formation acts as the primary nucleation mechanism for martensite during uniaxial tension in 304L stainless steel [41]. Under EBT conditions (Figure A1), a finer and more homogeneous grain structure is observed compared to the UT condition. The FSD images show lines and dark regions with increasing strain, but to a lesser extent than in the UT condition, indicating a more uniform distribution of strain. The BC images show that grain refinement occurs from the early stages of deformation, and the grain size remains relatively uniform up to 45% strain (ε = 0.45). This is consistent with the uniform distribution of martensite formation discussed in conjunction with Figure 11b. Ref. [21] reported that under equi-biaxial tension in 304 stainless steel, the strain is distributed more uniformly, suppressing shear band formation and leading to a finer and more homogeneous distribution of martensite.
